# Complex Impacts of PI3K/AKT Inhibitors to Androgen Receptor Gene Expression in Prostate Cancer Cells

**DOI:** 10.1371/journal.pone.0108780

**Published:** 2014-10-31

**Authors:** Liangliang Liu, Xuesen Dong

**Affiliations:** 1 The Vancouver Prostate Centre, Department of Urologic Sciences, University of British Columbia, Vancouver, Canada; 2 Department of Obstetrics and Gynaecology, University of Toronto, Toronto, Ontario, Canada; Florida International University, United States of America

## Abstract

**Background:**

Androgen deprivation therapy (ADT) is the first-line treatment to metastatic prostate cancer (PCa). However, sustained expression and function of the androgen receptor (AR) gene contribute to the progression of castration resistant prostate cancers (CRPC). Additionally, tumors can adapt the PI3K/AKT survival pathway to escape ADT. Co-targeting AR and PI3K/AKT signaling has been proposed to be a more effective therapeutic means for CRPC patients. Many clinical trials are ongoing to test whether PI3K/AKT inhibitors are beneficial to PCa patients. However whether these inhibitors have any impacts on the expressions of full length AR (AR-FL) and its splice variant (AR-V7) remains unclear.

**Methods:**

Four human prostate cancer cell lines (LNCaP, LNCaP95, VCaP and 22Rv1) with different genetic backgrounds were treated with five PI3K/AKT inhibitors (LY294002, Wortmannin, BKM120, AKTi and AZD5363) and or AKT siRNA. AR and AR-V7 protein and mRNA levels were measured by immunoblotting and real-time PCR assays. AR gene transcription initiation, alternative RNA splicing and AR mRNA degradation rates were also determined.

**Results:**

PI3K/AKT inhibitors had various impacts on AR protein expressions primarily through alterations of AR gene transcription initiation and RNA splicing. However, these effects remained unchanged in the presence RNA silencing of the AKT genes.

**Conclusion:**

PI3K/AKT inhibitors have off-target effects on AR gene expression in prostate cancer cells, which shall be considered when applying these inhibitors to PCa patients, particularly patients under ADT treatment.

## Introduction

Androgen deprivation therapy (ADT) is the standard treatment for metastatic prostate cancer (PCa). However, progression to castration resistant prostate cancer (CRPC) occurs to majority of patients [1]. CRPC tumours sustain the expression of AR and its regulated genes, indicating that the AR signaling continues to function [2]–[5]. Several mechanisms have been proposed for aberrant AR re-activation post ADT including: i) AR gene amplification and gain-of-function mutations [4], [6]–[9]; ii) alterations in expression and function of key AR co-regulators [10]–[12]; and iii) importantly, generation of ligand binding domain truncated AR splice variants (AR-Vs) [13]–[16] that constitutively activate the AR signaling. Among these variants, AR-V7 (also called AR3) is the most abundantly expressed AR-V in PCa [13], [14], [17]. AR-V7 protein levels are significantly elevated in CRPC tumors and closely associated with shorter patient survival [13], [14], [18]. These findings emphasize that blocking AR gene expression and function remains an important therapy.

Additionally, the phosphatidylinositol 3-kinase (PI3K)/AKT/mammalian target of rapamycin (mTOR) signaling is frequently activated in PCa and has been demonstrated to play important roles for CRPC progression and resistance to therapy-induced cell death [19], [20]. Genetic alterations of components of the PI3K/AKT/mTOR pathway occurred in 42% of primary prostate tumors and 100% of metastatic tumors [19]. More importantly, reciprocal feedback activation of AR and PI3K/AKT pathways had been demonstrated, which permits cancer cells to adapt either pathway for survival when the other is pharmacologically inhibited [21], [22]. These findings provide a rationale that co-targeting both pathways may achieve better outcomes for CRPC patients.

There are several inhibitors targeting different key components of the PI3K/AKT pathway including PI3K, AKT and mTOR. However, PI3K inhibitors such as LY294002 have also been demonstrated to bind and inhibit other kinases that do not belong to the PI3K/AKT signaling [23], [24]. In addition, studies have shown that when mTOR activity is inhibited by some AKT inhibitors, it can trigger a feedback mechanism resulting in re-activation of AKT or mitogen-activated proteins [25], [26]. Together, these findings indicated that beyond suppressing AKT downstream effectors, off-target effects of AKT inhibitors could produce profound impacts to cancer cells. The question remains to be answered is whether PI3K/AKT inhibitors can alter the expressions of full length AR (AR-FL) and AR-V7 in PCa cells, which could possibly counteract the effectiveness of ADT.

In this study, four PC cell lines were treated with five PI3K/AKT inhibitors. We measured both AR mRNA and protein levels and determined AR gene transcription initiation, RNA splicing and AR mRNA degradation rates. We reported there existed complex impacts of PI3K/AKT inhibitors to AR gene expression that are independent to AKT knockdown. These off-target effects on AR gene expression need to be considered when applying PI3K/AKT inhibitors to PCa patients.

## Materials and Methods

### Prostate cancer cell lines, PI3K/AKT inhibitors and siRNA transfection

LNCaP, VCaP and 22Rv1 human prostate cancer cell lines were obtained from the American Type Culture Collection (Manassas, VA). LNCaP cells were between 42–50 passages. LNCaP95 cell line was provided by Dr. Plymate (University of Washington) and was reported in previous studies [14], [27], [28]. It is derived from LNCaP cell and has obtained the resistance to androgen depletion conditions. Both LNCaP and LNCaP95 express mutant AR (T877A) that can activate AR by a broad range of steroids or steroid analogs. They also express mutant phosphatase and tensin homolog (PTEN) gene that constitutively activates AKT. VCaP cells express wild type AR and PTEN. But they possess AR gene amplification resulting in higher levels of AR-FL and AR-V7 expressions. 22Rv1 cells express wild type PTEN, but have gene rearrangement of the AR gene resulting high levels of AR-V7 expression. Together these four PCa cell lines represent a wide range of genetic alterations of PCa cells. LY294002 and Wortmannin were purchased from Cayman Chemicals (Ann Arbor, MI). NVP-BKM120 and AZD5363 were from Selleck Chemicals (Houston, TX). AKTi (CAT#124005) was from EMD Millipore (Billerica, MA). Control and AKT siRNAs (CAT#: J-003000, J-003001 and J-003002 for AKT 1–3 isoforms respectively) were from Dharmacon (Ottawa, Ontario). SiRNAs were transfected into PCa cells by using siLentFect Lipid Reagent from Bio-Rad (Mississauga, ON) according to the manufacturer's protocol.

### Immunoblotting assays

Protein lysates were collected with lysis buffer (50 mM Tris pH 8.0, 150 mM NaCl, 1% NP40, 0.5% sodium deoxycholate and 0.1% SDS) supplemented with protease and phosphatase inhibitors from Roche (Laval, QC) as we described [29]. Protein concentration of each sample was determined using the BCA kit from Pierce (Rockford, IL) per manufacturer's instructions. Protein samples were separated by SDS-PAGE gels, transferred to polyvinyl difluoride (PVDF) membranes and blotted with indicated antibodies. Experiments were repeated at least three times and one set of the representative blots was shown. Antibody information is listed in Table S1.

### Real-time PCR

Real-time PCR assays were performed as we described [30], [31]. Total RNA was extracted using Purelink RNA mini kit from Invitrogen (Burlington, ON) according to the manufacturer's instructions. Real-time PCR was conducted in triplicates using Applied Biosystems7900HT with 5 ng of cDNA, 1 µM of each primer pair and SYBR Green PCR master mix from Roche (Laval, QC). All real-time PCR assays were carried out using three technical replicates and three independent cDNA syntheses. Primer information is listed in Table S1.

### Nuclear run-on assay

Nuclear run-on assays were performed as described [32] with minor modifications. LNCaP and LNCaP95 cells were treated for 18 hours with vehicle, LY294002, AZD5363 or siRNA against AKT1–3 isoforms. Totally 6×10^6^ cells were washed with cold PBS, harvested and lysed on ice in 0.5% Nonidet P-40 lysis buffer [10 mM Tris·HCl (pH 7.4), 10 mM NaCl, 3 mM MgCl2] and centrifuged at 500×g for 10 min. Supernatants were removed and nuclei were incubated in reaction buffer [10 mM Tris·HCl (pH 8.0), 5 mM MgCl2, 0.3 mM KCl] containing 2.5 mM NTP plus Biotin-16-UTP mix from Roche (Laval, QC) for 45 min at 30°C. Biotinylated nascent RNA transcripts were precipitated by streptavidin beads from Gold Biotechnology (St. Louis, MO) and washed with Saline Sodium Citrate buffer plus 15% formaldehyde. Precipitated RNA was eluted from streptavidin beads and used as templates for real-time PCR analyses with primers amplifying mRNAs of total AR or 18S rRNA (18rS) as described in Table S1. Results were plotted as mean ± SEM from three independent repeats of each experimental condition.

### RNA splicing assay of the AR gene

Total RNA was extracted using Purelink RNA mini kit from Invitrogen (Burlington, ON). Real-time PCR was conducted to measure AR gene RNA splicing. The relative AR-FL and AR-V7 splicing efficiency was determined by normalization with an internal PCR product within AR exons 2 and 3. Primer information was listed in Table S1. All real-time PCR assays were carried out using three technical replicates and three independent cDNA syntheses.

### Statistics

Results are expressed as the mean ± SEM. To determine the differences among experimental groups, one-way ANOVA followed by student *t*-test was carried out using GraphPad Prism (version 4) with the level of significance set at P<0.05 as *, P<0.01 as ** and P<0.001 as ***.

## Results

### Impacts of PI3K/AKT inhibitors to AR protein levels in PCa cells

**Figure 1:**
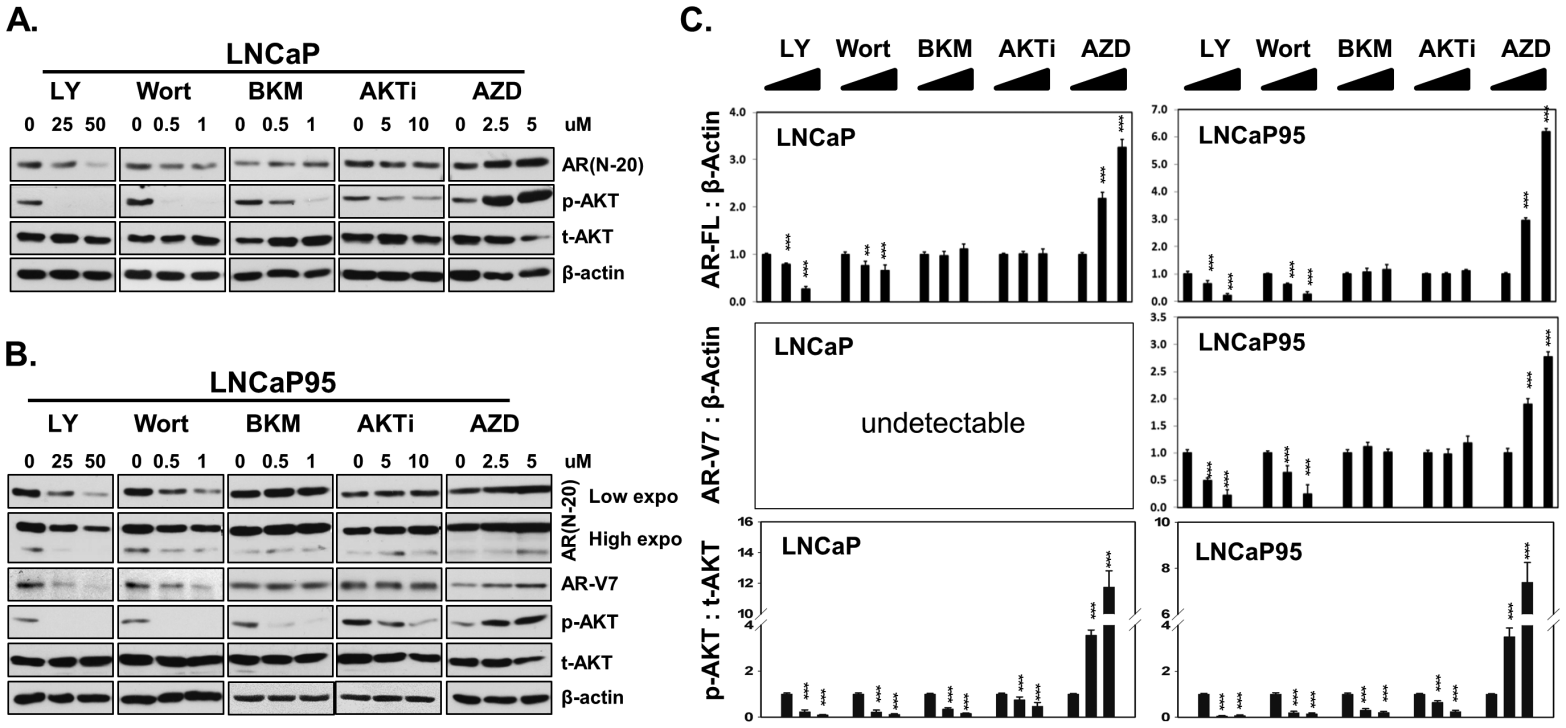
Impacts of PI3K/AKT inhibitors to AR protein levels in LNCaP and LNCaP95 cells. (**A**) LNCaP and (**B**) LNCaP95 cells were treated with increasing doses of PI3K/AKT inhibitors LY294002 (0, 25, 50 uM), Wortmannin (0, 0.5 and 1 uM), BKM120 (0, 0.5 and 1 uM), AKTi (0, 5 and 10 uM) or AZD5363 (0, 2.5 and 5 uM) for 18 hours. Protein lysates were immunoblotted with AR (N-20), AR-V7, Pan-AKT, phosphor-AKT (ser473) and β-Actin antibodies. (**C**) Results were repeated at least three independent experiments. Densitometry analysis of protein bands were measured by the Image J software and plotted as mean+SEM. One-way ANOVA followed by student t-test was performed with * as P<0.05, ** as P<0.01 and *** as P<0.001.

**Figure 2:**
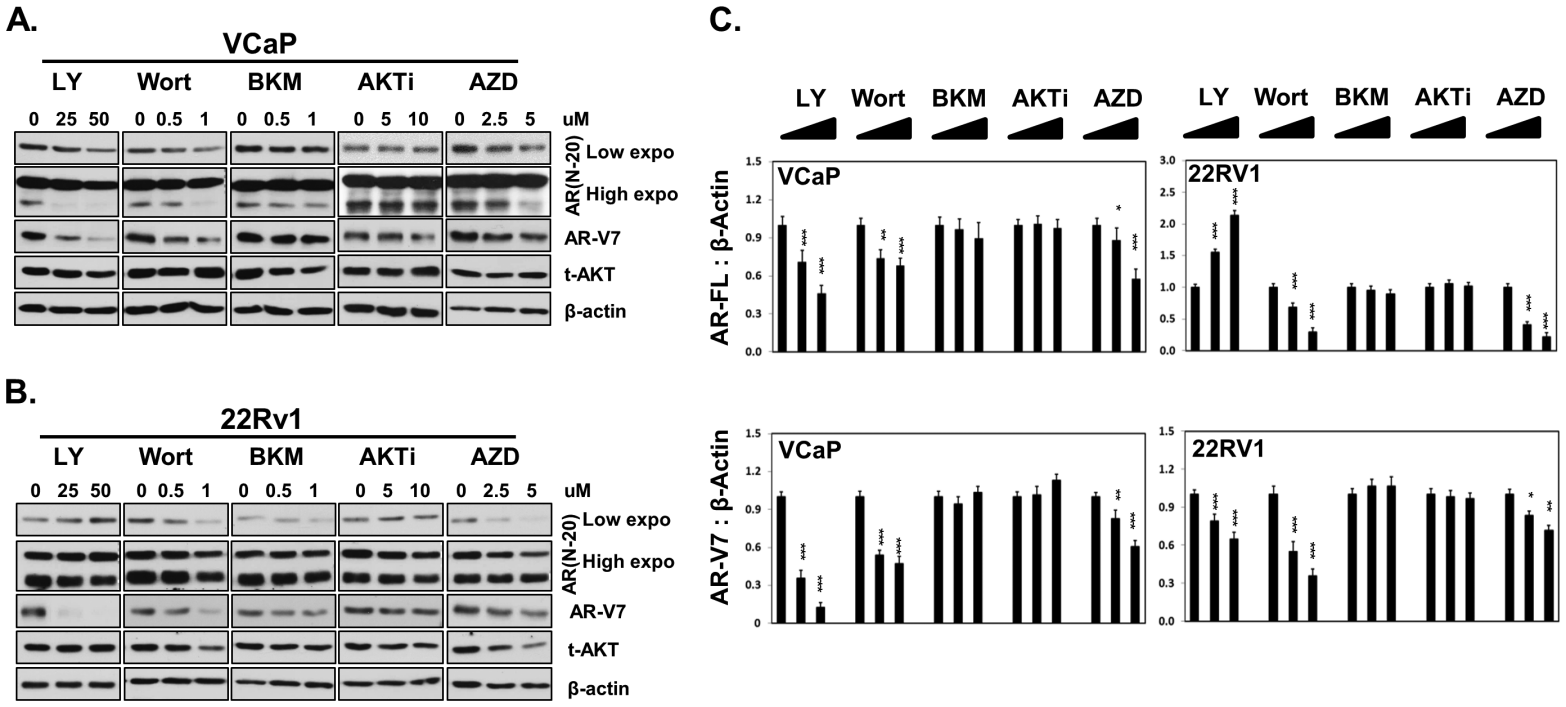
Impacts of PI3K/AKT inhibitors to AR protein levels in VCaP and 22Rv1. (**A**) VCaP and (**B**) 22Rv1 cells were treated with increasing doses of PI3K/AKT inhibitors LY294002 (0, 25, 50 uM), Wortmannin (0, 0.5 and 1 uM), BKM120 (0, 0.5 and 1 uM), AKTi (0, 5 and 10 uM) or AZD5363 (0, 2.5 and 5 uM) for 18 hours. Protein lysates were immunoblotted with AR (N-20), AR-V7, Pan-AKT, phosphor-AKT (ser473) and β-Actin antibodies. (**C**) Results were repeated at least three independent experiments. Densitometry analysis of protein bands were measured by the Image J software and plotted as mean+SEM. One-way ANOVA followed by student t-test was performed with * as P<0.05, ** as P<0.01 and *** as P<0.001.

LY294002 is a strong competitive inhibitor to PI3K and prevents PI3K from phosphorylating and activating downstream effectors of AKT [33]. Wortmannin is a covalent inhibitor of PI3K that irreversibly suppresses AKT phosphorylation [34]. LY294002 repressed AR-FL protein levels in LNCaP cells (Fig. 1A), inhibited both AR-FL and AR-V7 protein levels in LNCaP95 cells (Fig. 1B). Both LNCaP and LNCaP95 cells are PTEN deficient cells, in which AKT is constitutively active in its phosphorylation form (p-AKT). Both LY294002 and Wortmannin strongly inhibited p-AKT levels in both cell lines. Densitometry analyses on protein expressions of AR-FL, AR-V7 and p-AKT were measured (Fig. 1C). Interestingly, both LY294002 and Wortmannin suppressed AR-FL and AR-V7 protein expressions in VCaP cells (Fig. 2A). However, LY294002 increased AR-FL, but decreased AR-V7 protein levels in 22Rv1 cells (Fig. 2B). Wortmannin inhibited both AR-FL and AR-V7 protein expressions in VCaP and 22Rv1 cells. Densitometry analyses on protein expressions of AR-FL and AR-V7 were analyzed (Fig. 2C). Since VCaP and 22Rv1 cells are PTEN sufficient cells, where p-AKT is at very low/undetectable levels unless its upstream tyrosine kinase receptors are activated, the effects of LY294002 to AR protein expression in VCaP and 22Rv1 cells were not likely related to AKT activation. BKM120 is a pan-PI3K inhibitor, but not to mTOR [35]. BKM120 efficiently suppressed p-AKT levels in both LNCaP and LNCaP95 cells. However, BKM120 had no effects to AR-FL and AR-V7 protein levels in all four PCa cell lines (Fig. 1–2). AKTi is a competitive phosphatidylinositol ether analog that docks into the pleckstrin homolgy (PH) domain of AKT, prevents AKT translocation to PI3K at the plasma membrane thus inhibits AKT phosphorylation and activation [36]. AKTi did not show any suppressive effects to AR-FL or AR-V7 protein levels (Fig. 1–2). AZD5363 is an ATP-competitive inhibitor of AKT [37], [38]. It increased both AR-FL and AR-V7 protein levels and induced p-AKT levels in LNCaP and LNCaP95 cells (Fig. 1). However, it decreased AR-FL and AR-V7 protein levels in PTEN sufficient VCaP and 22Rv1 cells (Fig. 2).

### Impacts of PI3K/AKT inhibitors to AR mRNA levels in PCa cells

**Figure 3:**
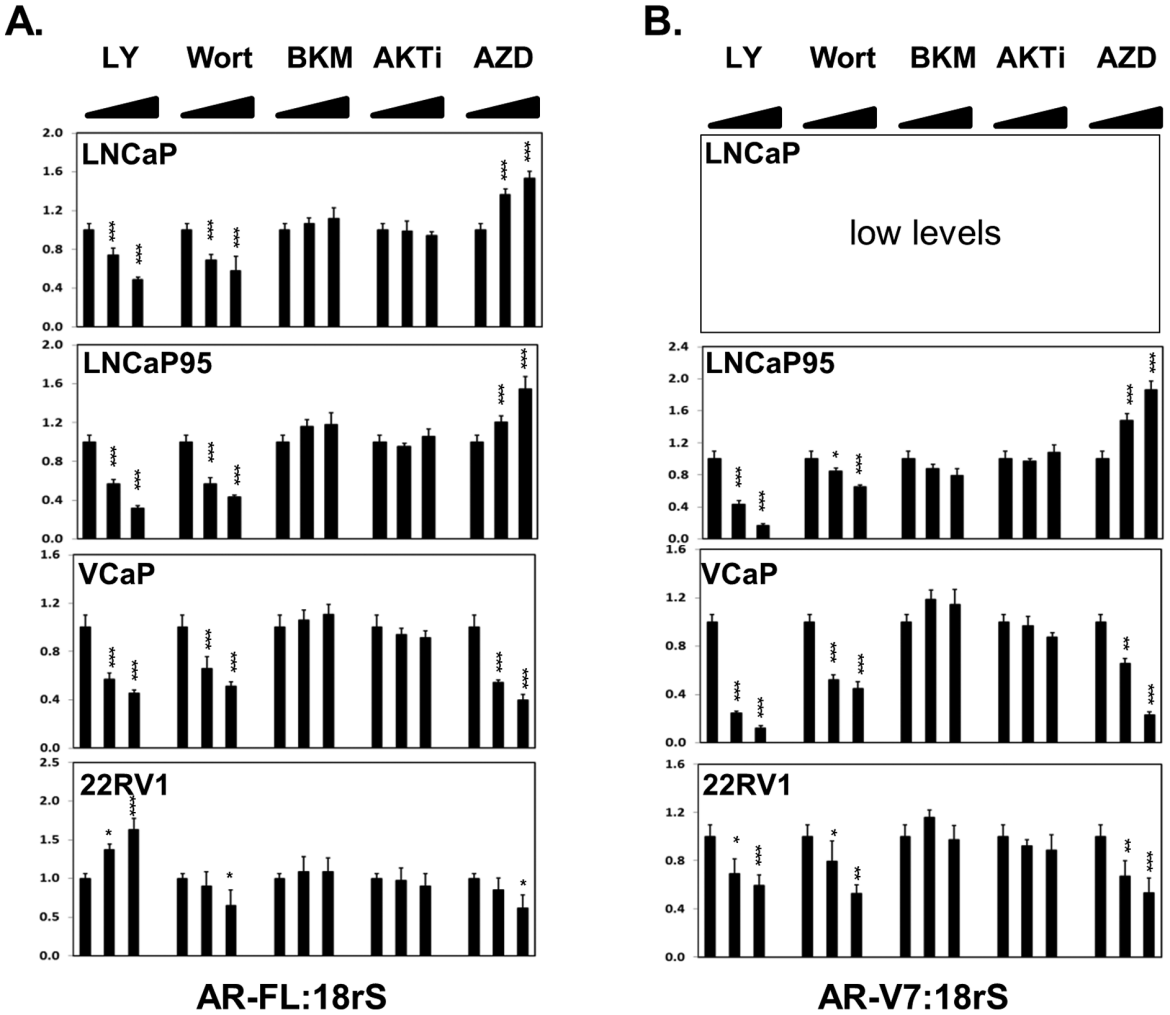
Impacts of PI3K/AKT inhibitors to AR mRNA levels in PCa cells. LNCaP, LNCaP95, VCaP and 22Rv1 cells were treated with increasing doses of PI3K/AKT inhibitors LY294002 (0, 25, 50 uM), Wortmanin (0, 0.5 and 1 uM), BKM120 (0, 0.5 and 1 uM), AKTi (0, 5 and 10 uM) or AZD5363 (0, 2.5 and 5 uM) for 18 hours. AR-FL (**A**) and AR-V7 (**B**) mRNA expression levels relative to 18rS were determined by real-time PCR. Results were from three independent RNA extractions with triplicate real-time PCR assays on each RNA sample. Data was plotted as mean+SEM. One-way ANOVA followed by student t-test was performed with * as P<0.05, ** as P<0.01 and *** as P<0.001.

AR mRNA levels were also measured in PCa cell lines under treatments of PI3K/AKT inhibitors. The changes of AR-FL and AR-V7 mRNA levels (Fig. 3) were consistent to what were observed in AR protein levels as shown in Figure 1 and 2. LY294002 and Wortmannin decreased both AR-FL and AV-7 mRNA levels in all PCa cell lines except that LY294002 increased AR-FL mRNA levels in 22Rv1 cells. BKM120 and AKTi had no significant impacts in AR mRNA levels. AZD5363 increased AR-FL and AR-V7 mRNA levels in LNCaP and LNCaP95 cells, but decreased their mRNA levels in VCaP and 22Rv1 cells with statistical significance. These results suggested that PI3K/AKT inhibitors affected AR gene expression primarily at mRNA level.

### Impacts of AKT knockdown to AR gene expression

**Figure 4:**
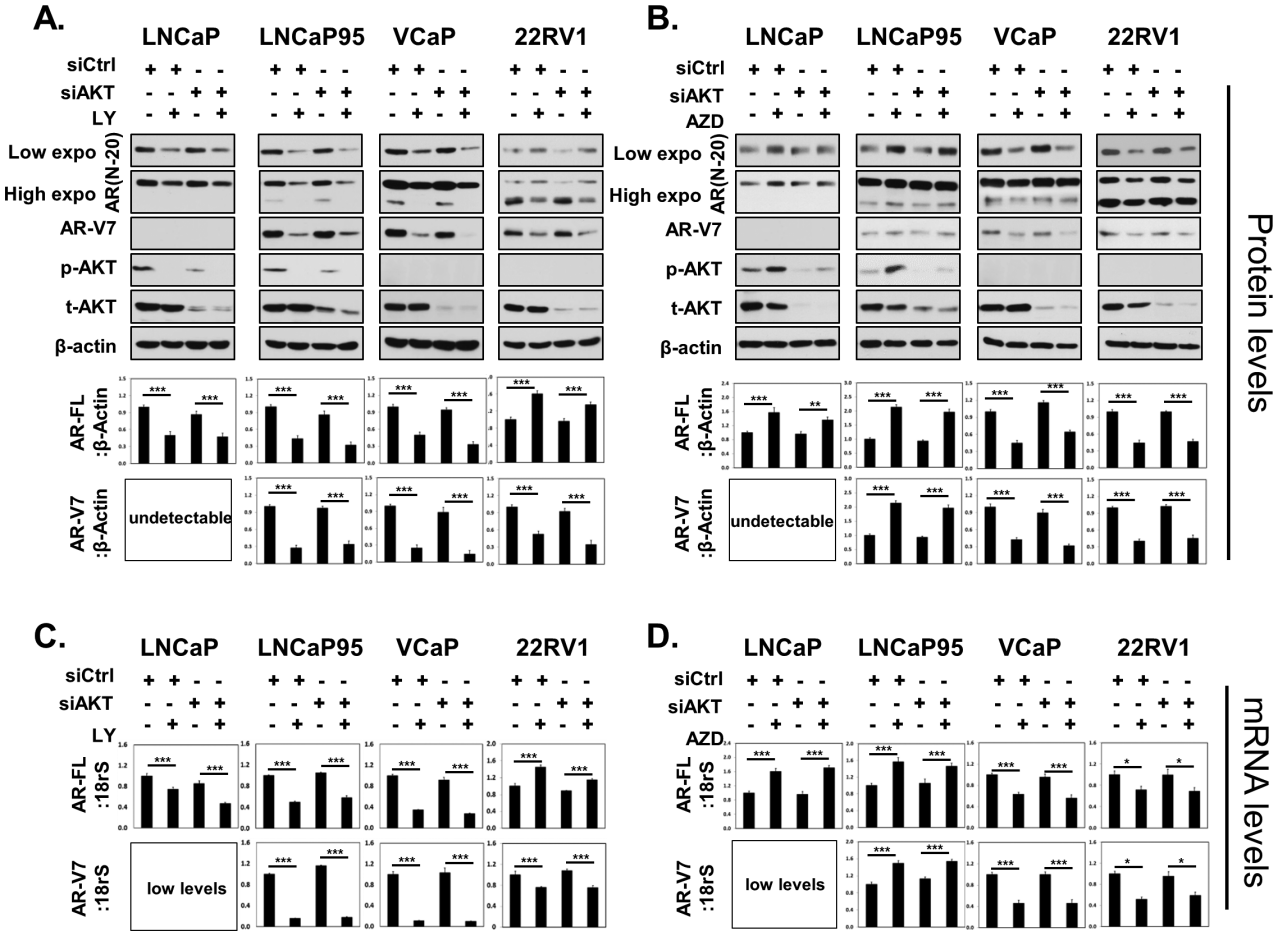
Impacts of AKT knockdown to AR gene expression. LNCaP, LNCaP95, VCaP and 22Rv1 cells were transfected with control or pooled siRNA for AKT 1–3 isoforms for 36 hours. Cells were then further treated with 50 uM LY294002 (**A**) or 5 uM AZD5363 (**B**) for another 18 hours. Protein lysates were immunoblotted with AR (N-20), AR-V7, Pan-AKT, p-AKT(ser473) and β-Actin antibodies. Results were repeated in more than three independent experiments. Densitometry analysis of protein bands were measured by the Image J software and plotted as mean+SEM. (**C**) AR-FL and (**D**) AR-V7 mRNA expression levels relative to 18rS were determined by real-time PCR. Results were from three independent RNA extractions with triplicate real-time PCR assays on each RNA sample and plotted as mean+SEM. Student's t-tests were performed comparing between vehicle and LY294002/AZD5363 treatments with * as P<0.05; ** as P<0.01 and *** as P<0.001.

To further investigate whether the impacts of LY294002 and AZD5363 on AR protein and mRNA levels were mediated through AKT, we performed AKT knockdown assays using pooled siRNA against all three AKT1–3 isoforms. Efficiency of AKT siRNA was shown by Western blotting (Fig. 4A–B). We observed that regardless the presence of AKT knockdown, LY294002 can still dramatically decrease AR-FL and AR-V7 protein (Fig. 4A) and mRNA (Fig. 4C) levels in LNCaP, LNCaP95 and VCaP cells. LY294002 upregulated AR-FL expression, but decreased AR-V7 expression in 22Rv1 cells. In addition, we observed that AZD5363 significantly increased AR-FL expressions in LNCaP and LNCaP95 cells and decreased AR-FL and AR-V7 in VCaP and 22Rv1 cells, which effects remained unchanged even in the presence of efficient AKT knockdown (Fig. 4B and 4D). Together, these results indicated that the impacts of LY294002 and AZD5363 to AR expressions were independent to AKT and its downstream effectors.

### LY294002 and AZD5363 affect AR gene transcription initiation, RNA splicing and AR mRNA stability

**Figure 5:**
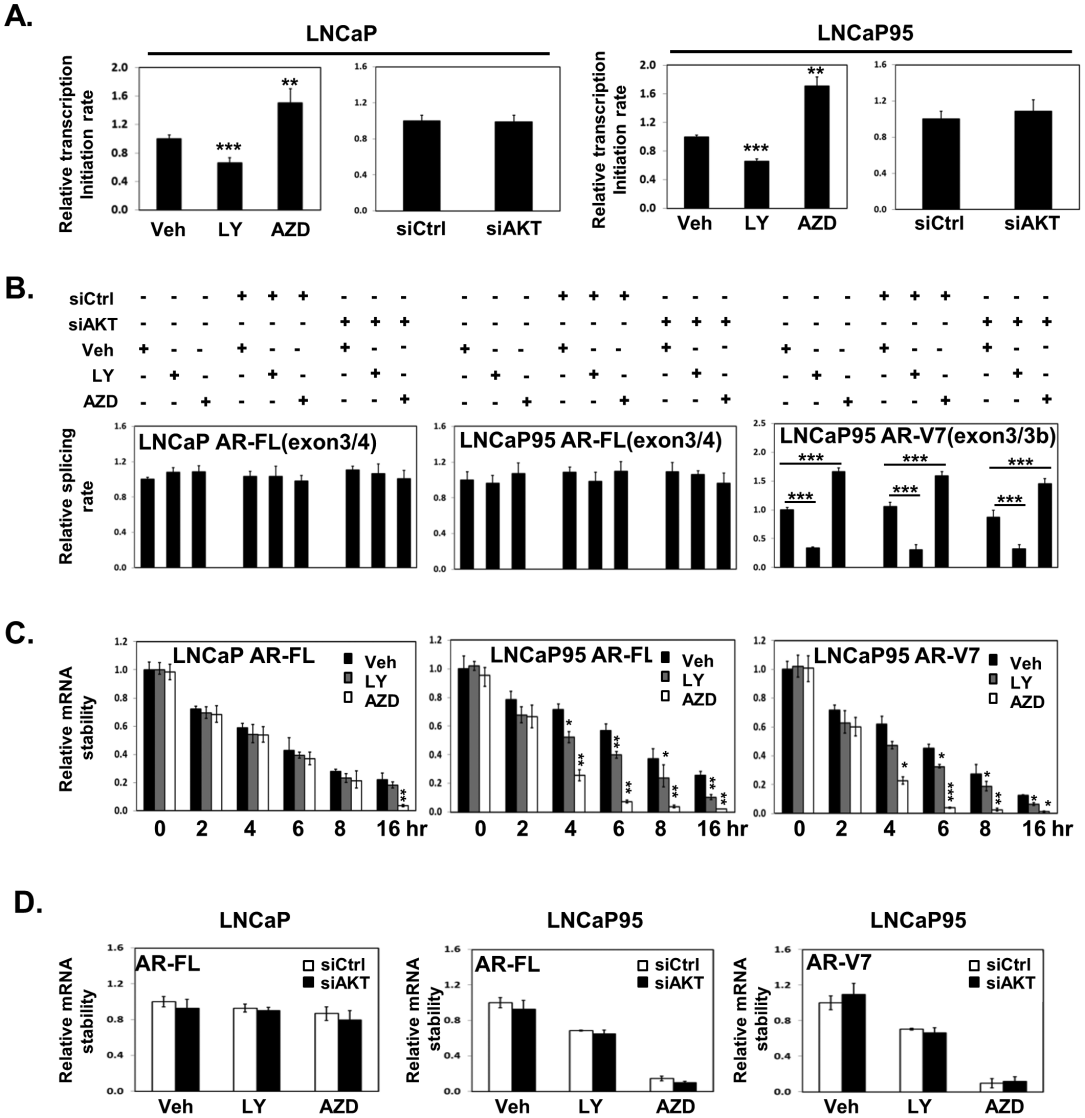
LY294002 and AZD5363 affect AR gene transcription initiation, RNA splicing and AR mRNA stability. (**A**) LNCaP and LNCaP95 cells were treated with vehicle, 50 uM LY294002 or 5 uM AZD5363 for 18 hours (left). LNCaP and LNCaP95 cells were also transfected with control or AKT1–3 siRNAs for 48 hours (right). Nuclear run-on assays were performed as described in Material and Method section. (**B**) LNCaP and LNCaP95 cells were transfected with control or pooled siRNA for AKT 1–3 isoforms for 36 hours. Cell were then further treated with 50 uM LY294002 or 5 uM AZD5363 for another 18 hours. RNA splicing assays for AR-FL and AR-V7 were measured as described in Material and Method section. (**C**) LNCaP and LNCaP95 cells were pretreated with 2 uM actinomycin D for 2 hours. Cells were then treated with vehicle, 50 uM LY294002 or 5 uM AZD5363 for 0, 2, 4, 6, 8, and 16 hours. AR-FL and AR-V7 mRNA expression levels relative to 18rS were determined by real-time PCR. (**D**) LNCaP and LNCaP95 cells were transfected with control or pooled siRNA for AKT 1–3 isoforms for 36 hours. Cell were pre-treated with 2 uM Actinomycin D for 2 hours and then treated with vehicle, 50 uM LY294002 or 5 uM AZD5363 for 6 hours. AR-FL and AR-V7 mRNA expression levels relative to 18rS were determined by real-time PCR. Results were from three independent RNA extractions with triplicate real-time PCR assays on each RNA sample. One-way ANOVA followed by student t-test was performed with * as P<0.05, ** as P<0.01 and *** as P<0.001.

The impacts of LY294002 and AZD5363 on AR mRNA levels could be at multiple possible levels including AR gene transcription initiation, pre-mRNA splicing and AR mRNA degradation. Using nuclear run-on assay, we measured both AR and 18rS gene transcription initiation rates in presence of vehicle or PI3K/AKT inhibitors (Fig. 5A). The 18rS gene was used as an internal control as its mRNA levels were not affected by PI3K/AKT inhibitors. We showed that LY294002 inhibited, while AZD5363 enhanced AR gene transcription initiation rates relative to 18rS in both LNCaP and LNCaP95 cells. Furthermore, knocking down AKT did not alter AR gene transcription rates.

To measure the *bona fide* effects of LY294002 and AZD5363 on AR-FL and AR-V7 RNA splicing, but exclude their impacts on AR gene transcription initiation, we designed three sets of primers to measure the relative splicing rates for AR-FL and AR-V7. Since AR-V7 RNA splicing is a mutually exclusive RNA splicing process of exon 3b and exon 4 of the AR pre-mRNA, one set of primers cover the exon 3 and 4 junction of the AR gene for measuring AR-FL mRNA levels, one set of primers cover exon 3 and 3b for AR-V7 mRNA levels and the other set of primers are within exons 2 and 3 for total AR mRNA levels. We showed that AR-FL RNA splicing was not affected by LY294002, AZD5363 or AKT knockdown. However, LY294002 dramatically inhibited, while AZD5363 increased AR-V7 splicing in LNCaP95 cells (Fig. 5B). The changes of AR-V7 splicing induced by LY294002 or AZD5363 were not altered in the presence of AKT knockdown.

AR-FL and AR-V7 mRNA stabilities were also studied in LNCaP and LNCaP95 cells pre-treated with actinomycin D (Fig. 5C). LY294002 enhanced AR-FL and AR-V7 mRNA degradation in LNCaP95 cells within 16 hours. AZD5363 enhanced AR-FL and or AR-V7 mRNA degradation in both LNCaP and LNCaP95 cells. However, the impacts of LY294002 and AZD5363 on AR mRNA stabilities were not affected by AKT knockdown (Fig. 5D). Since the total mRNA levels of AR-FL and AR-V7 in LNCaP and LNCaP95 cells were increased by AZD5363 (Fig. 3), these results indicated that the impacts of AZD5363 on AR gene transcription initiation and RNA splicing overweighed its effects to AR mRNA degradation.

### LY294002 and AZD5363 affect AR-FL and AR-V7 transcriptional activity

**Figure 6:**
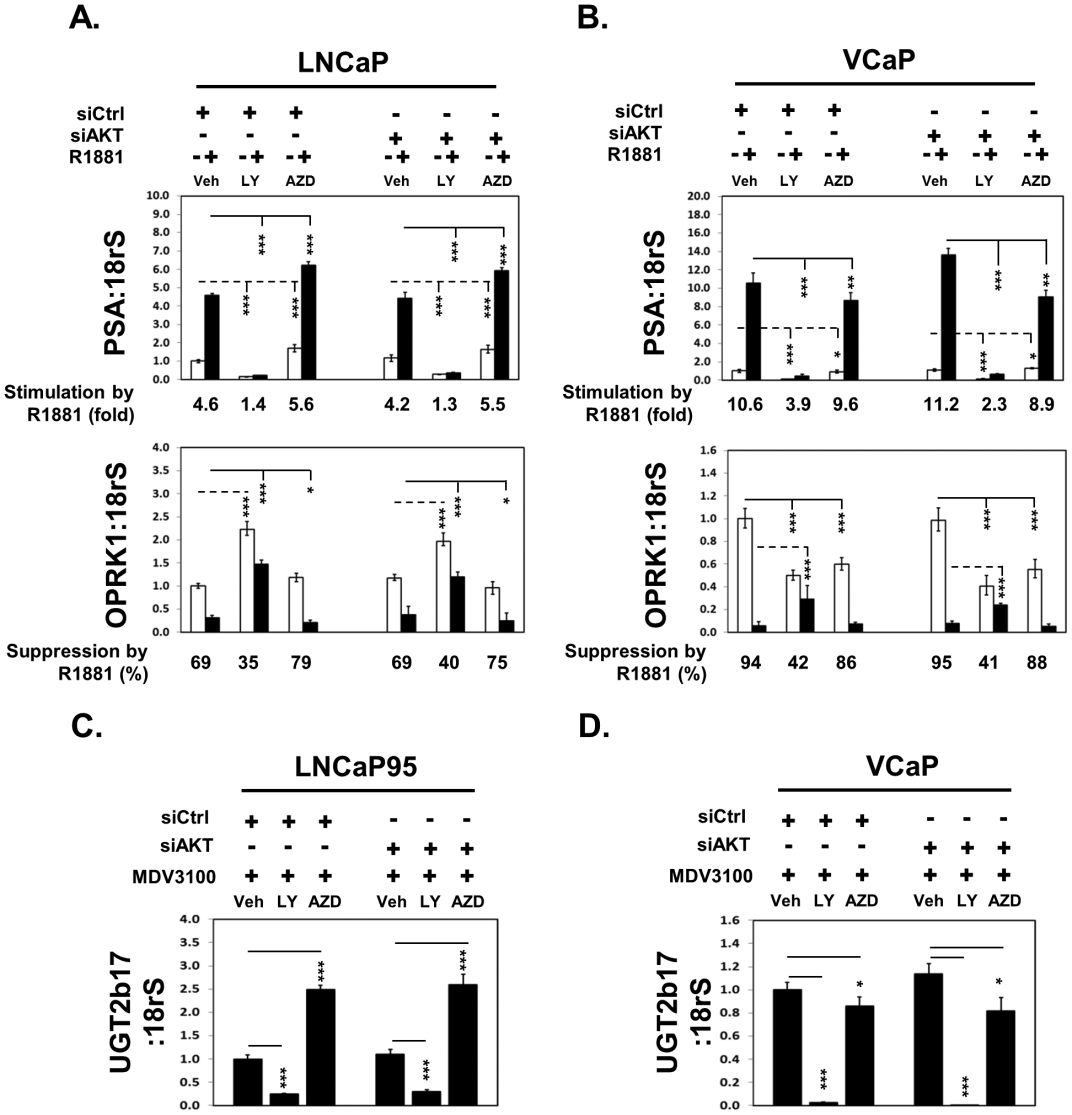
LY294002 and AZD5363 affect AR and AR-V7 transcriptional activity in PCa cells. (**A**) LNCaP and (**B**) VCaP cells were transfected with control or AKT1–3 siRNAs for 48 hours and treated with vehicle or 1 nM R1881. Cells were also co-treated with vehicle, 50 uM LY294002 or 5 uM AZD5363 for 18 hours. Real-time PCR assays measured PSA and OPRK1 mRNA levels. (**C**) LNCaP95 and (**D**) VCaP cells were transfected with control or AKT1–3 siRNAs for 48 hours and treated with vehicle, 50 uM LY294002 or 5 uM AZD5363 in the presence of 5 uM MDV3100 for 18 hours. Real-time PCR assays measured UGT2b17 mRNA levels. Results were from three independent RNA extractions with triplicate real-time PCR assays on each RNA sample. Student's t-tests were performed comparing between vehicle and LY294002/AZD5363 treatments with * as P<0.05; ** as P<0.01 and *** as P<0.001.

To further investigate whether alterations of AR-FL and AR-V7 protein levels by LY294002 and AZD5363 would impact AR transcriptional activities, we measured the expressions of two AR-FL regulated genes (PSA and OPRK1) and one AR-V7 regulated gene (UGT2b17). In both LNCaP and VCaP cells, AR-FL agonist R1881 stimulated PSA expression, which actions were suppressed by LY294002 (Fig. 6A–B). By contrast, AZD5363 strengthened the R1881 effect in upregulating PSA expression in LNCaP cells, but reduced the R1881 action in VCaP cells. These changes were consistent to the impacts of LY294002 and AZD5363 on AR-FL protein levels in these cells. The expression of OPRK1 gene was suppressed by ligand activated AR-FL [39]. Treatment of LY294002 decreased AR-FL mediated inhibition to OPRK1 expression in both LNCaP cells (69% suppression reduced to 35%) and VCaP cells (94% suppression reduced to 42%). AZD5363 strengthened AR-FL in suppressing OPRK1 expression in LNCaP cells (69% suppression increased to 79%), but reduced AR-FL mediated inhibition of OPRK1 expression in VCaP cells (94% suppression reduced to 86%). However, both impacts of LY294002 and AZD5363 on PSA and OPRK1 expressions were not significantly altered by AKT knockdown. To study the AR-V7 transcriptional activity, we measured the expression of UGT2b17 in VCaP and LNCaP95 cells that were treated with MDV3100 to block AR-FL activity. Both VCaP and LNCaP95 cells express both AR-FL and AR-V7. We have previously demonstrated that in the presence of AR-FL functional blockade, the upregulation of expression of UGT2b17 was determined by AR-V7 [27]. LY294002 inhibited UGT2b17 expression in both LNCaP95 and VCaP cells (Fig. 6C–D). AZD5363 stimulated UGT2b17 expression in LNCaP cells, but reduced its expression in VCaP cells. These changes in UGT2b17 gene expression were consistent to AR-V7 protein levels under LY294002 and AZD5363 treatments. However, these impacts were not altered by AKT knockdown.

## Discussion

Aberrant activation of the PI3K/AKT pathway through either genetic or epigenetic mechanisms frequently occurs in tumors, rendering this pathway an attractive therapeutic target for a wide spectrum of cancers. Many inhibitors are available to block PI3K/AKT signaling and some are under early clinical trials. However, heterogeneity of prostate cancers is a special feature, which makes it difficult to anticipate whether inhibition of PI3K/AKT signaling would benefit PCa patients. Additionally, many possible biological sequelae of PI3K/AKT inhibition may further undermine the potential therapeutic gain. This study aims to investigate whether PI3K/AKT inhibitors have any off-target effects to AR gene expression in a panel of PCa cell lines that represent prostate tumor cells with a broad range of genetic variations. It is important to document the expression profile of AR-FL and AR splice variants under treatments of PI3K/AKT inhibitors, since sustained AR expression and function are one of the critical molecular mechanisms by which prostate tumors progress into CRPC. We reported that PI3K/AKT inhibitors had complex impacts on AR gene expression that were independent to AKT, suggesting that these PI3K/AKT inhibitors could affect signaling beyond the PI3K/AKT pathway in PCa cells pending upon their genetic backgrounds. These off-target effects cautioned potential application of PI3K/AKT inhibitors to certain, if not all, PCa patient populations.

Off-target effects of PI3K/AKT inhibitors had been well documented and repeatedly observed in this study. LY294002 and Wortmannin were demonstrated to bind many other kinases that were not related to PI3K/AKT signaling [23], [24]. It is therefore not surprising that LY294002 and Wortmannin had shown dominant negative effects to AR gene expression even under AKT knockdown condition. Although both BKM120 and AKTi dramatically suppressed AKT phosphorylation and activation, they did not show any impacts to AR gene expressions in all four PCa cell lines. These results excluded the possibility that activation of AKT was directly responsible for changes of AR gene expressions. They served as negative controls to further support the off-target effects of LY294002, Wortmannin and AZD5363 on AR gene expressions. Interestingly, AZD5363 can alter AR-FL and AR-V7 expressions pending upon the genetic background of PCa cell lines. However, several evidences ubiquitously indicated the off-target effects of AZD5363 to AR gene expression. First, although LNCaP and LNCaP95 cells express mutant PTEN and constitutively active AKT, AZD5363 increased both AR-FL and AR-V7 expressions even when endogenous AKT were significantly depleted by RNA silencing (Fig. 4). These results indicated that the feedback activation of AKT by AZD5363 did not contribute to the changes of AR expressions. Second, VCaP and 22Rv1 cells express wild type PTEN with low/undetectable levels of p-AKT in the absence of activation of the upstream tyrosine kinase receptors (Fig. 1–2). The impacts of AZD5363 in reducing AR-FL and AR-V7 in VCaP and 22Rv1 lines were thus not related to AKT activation. Last, AZD5363 can enhance AR gene transcription initiation (Fig. 5A), RNA splicing of AR-V7 (Fig. 5B), AR-FL and AR-V7 mRNA degradation independent to AKT knockdown (Fig. 5C–D).

Both AR and PI3K/AKT signaling had been demonstrated to be important for PCa cells to develop therapy resistance. Development of inhibitors to these signaling provides new opportunities for PCa patients. The counterbalance of these opportunities is the challenge of intrapatient PCa cell heterogeneity [40]. Multiple clones of cancer cells with different genetic background could co-exist in the same patient [41]. Therefore one PI3K/AKT inhibitor could be effective to one population of cancer cells, but possibly provide growth privilege for others through clonal selection process. Co-targeting AR and PI3K/AKT pathways can be even more challenging. Reciprocal activation of AR and PI3K/AKT signaling in prostate cancers could complicate the outcome of PCa patients receiving combinational treatments of AR and PI3K/AKT inhibition. Anti-androgens can possibly undermine the effectiveness of PI3K/AKT inhibitors. Vice versa, PI3K/AKT inhibitors may counteract the effectiveness of anti-androgens. Recent studies using PTEN knockout mouse models further showed that although castration sensitive tumors replied on AR and PI3K/AKT signaling, the derived castration resistant tumors undergo phenotypic plasticity leading to increased intratumoral heterogeneity and loss of tumor independence to PI3K signaling [42]. Our previous studies had shown that castration conditions induce AR gene transcription and RNA splicing of AR-V7 [27]. We further showed here that inhibitors such as AZD5363 can increase AR gene transcription initiation and AR-V7 RNA splicing. Co-treatment of anti-androgen and AZD5363 would possibly result in enhanced AR-V7 expression and promote AR-V7 driven tumors in a subgroup of PCa patients.

In summary, our studies demonstrated PI3K/AKT inhibitors have various off-target effects on AR gene expressions in PCa cells with different genetic backgrounds. This information should be considered when treating PCa patients with these inhibitors.

## Supporting Information

Table S1 **Information of antibodies and primers used in this study.**
(DOCX)
