# Supplementary material for: Complex Impacts of PI3K/AKT Inhibitors to Androgen Receptor Gene Expression in Prostate Cancer Cells
Source: PLoS One. 2014 Oct 31;9(10):e108780. doi: 10.1371/journal.pone.0108780 (PMC4215833; doi:10.1371/journal.pone.0108780)
Supplement: Table S1 — Information of antibodies and primers used in this study. (DOCX) [file pone.0108780.s001.docx]

**Table S1**

**Antibodies information**

| **Name** | **Clone** | **Company(CAT#)** |
| --- | --- | --- |
| AR | N-20 | Santa Cruz (sc-816) |
| AR-V7 |  | Presion antibody (AG10008) |
| pan-AKT | C67E7 | Cell signaling (#4691) |
| phosphor-AKT (Ser473) |  | Cell signaling (#9271) |
| β-actin | AC-15 | Sigma-Aldrich (A5441) |

**Real-time PCR Primers information**

| **Name** | **Primer sequence** | **Location** |
| --- | --- | --- |
| AR-V7 F | 5'-CAGGGATGACTCTGGGAGAA-3' | Exon 3/3b |
| AR-V7 R | 5'-GCCCTCTAGAGCCCTCATTT-3' | 3’ UTR |
| AR-FL F | 5'-TCTTGTCGTCTTCGGAAATGT-3' | Exon 3 |
| AR-FL R | 5'-AAGCCTCTCCTTCCTCCTGTA-3' | Exon 4 |
| AR Total F | 5'-GTGGAAGCTGCAAGGTCTTC -3' | Exon 2 |
| AR Total R | 5'-GGCGCACAGGTACTTCTGTT-3' | Exon 3 |
| 18S rRNA F | 5′-TTGACGGAAGGGCA CCACCAG-3′ |  |
| 18S rRNA R | 5′-GCACCACCACCCACGGAATCG-3’ |  |
| PSA F | 5'-AGTGCGAGAAGCATTCCCAAC -3' |  |
| PSA R | 5'-CCAGCAAGATCACGCTTTTGTT -3' |  |
| OPRK1 F | 5'-AACTCGCTGGTCATGTTCGT -3' |  |
| OPRK1 R | 5'-CTCTGAAAGGGCATGGTTGT -3' |  |
| UGT2b17 F | 5'-TGACTTTTGGTTTCAAGC-3' |  |
| UGT2b17 R | 5'-TTCCATTTCCTTAGGCAA-3' |  |
|  |  |  |
